# What Matters Most for Community Social Capital among Older Adults Living in Urban China: The Role of Health and Family Social Capital

**DOI:** 10.3390/ijerph16040558

**Published:** 2019-02-15

**Authors:** Jingyue Zhang, Nan Lu

**Affiliations:** 1 Department of Sociology, School of Philosophy and Sociology, Jilin University, Changchun 130012, China; zjyz13@mails.jlu.edu.cn; 2 Institute of Gender and Culture, Changchun Normal University, Changchun 130052, China; zhangjingyue@mail.cncnc.edu.cn; 3 Department of Social Work, School of Sociology and Population Studies, Renmin University of China, Beijing 100872, China

**Keywords:** cognitive social capital, structural social capital, determinants, China

## Abstract

The present study investigated individual-level determinants of community social capital among older adults in urban China, with a particular emphasis on health and family social capital. A quota sampling method was used to select 456 adults aged 60 or older from 16 local communities in the city of Suzhou in 2015. Multiple indicators and multiple courses in structural equation modeling were used to examine the proposed model. Latent constructs of community social capital (i.e., cognitive social capital and structural social capital) were established. The results showed that family social capital and instrumental activities of daily living were the most influential determinants of cognitive social capital, whereas activities of daily living and socioeconomic status were the most important determinants of structural social capital. We demonstrate the application of social capital theory in an urban Chinese context. Future policy development and social work interventions should use a more comprehensive social capital latent constructs and health indicators as screening instruments. The promotion of family social capital could play an important role in enhancing cognitive social capital among older adults.

## 1. Introduction

Social capital is an important social determinant of well-being among older populations across countries and cultures. Many studies have focused on the consequences of social capital [1]. For example, social capital was found to have important effects on self-rated health (SRH), life satisfaction, depression, physical disease, and even mortality among older adults [1,2,3,4,5]. However, studies on the determinants of social capital among older populations are limited. Many studies have controlled for a range of sociodemographic variables (e.g., age, gender, marital status, education) when testing the relationship between social capital and health [2,6]. However, there is a lack of research that can explain the associations between these variables and social capital. The findings of such studies would enhance the understanding of what matters for social capital, and would have important implications for policy and interventions aimed at improving the health of older populations through promoting social capital.

Many studies use a single indicator to represent the multidimensional concept of social capital. Different studies also tend to use different methods of assessing social capital [7]. Therefore, it is difficult to compare findings among studies and generate meaningful conclusions. Moreover, social capital has different meanings and functions across different life stages [1]. However, many studies on social capital draw their samples from the general population. Studies on the determinants of social capital in later life are limited. Furthermore, the majority of relevant studies have examined the effects of social capital on a range of health outcomes from the social causation perspective. However, the above relationships can be bidirectional, and the social selection model offers an alternative explanation [8]. Compared with those with disabilities and severe illnesses, individuals who are free of health problems might be more likely to actively participate in both informal and formal social activities in local communities, and develop higher levels of community social capital. 

Finally, social capital is culturally sensitive. Empirical evidence shows that ethnicity, migration, and historical background could explain variations in levels of social capital [9]. There is a lack of research on what matters for social capital in China, where familism and Confucian ideology are greatly emphasized. Family and community are two of the most important sources of social capital for older adults in China [10,11]. However, the interplay between social capital indicators embedded from family and community systems have been largely unstudied (hereafter community social capital and family social capital). Therefore, the present study aimed to fill this gap in the research by building and testing latent constructs of community social capital, and examined the social determinants of community social capital in an urban Chinese context, with a particular emphasis on health and family social capital. 

### 1.1. Defining Social Capital 

Experts in different research fields have defined social capital from different perspectives. The most adopted definition is from the perspective of social cohesion, which defines social capital as “features of social organization, such as trust, norms, and networks that can improve the efficiency of society by facilitating coordinated action” [12]. Social capital can also be viewed as social resources embedded from individuals’ social relationships in their local communities and/or family systems, where they have shared norms, values, and common memberships [13]. Lin [14] examined individuals’ investment in their social networks and how individuals invest their social supportive resources with expected returns. While previous researchers paid close attention to the closure or density of social networks, Lin proposed that bridges in social connections also play an important role in sharing information and knowledge, and searching for important social resources from other social networks. Furthermore, there is a lack of consensus on the measurement levels and instruments on social capital. Social capital can be measured from individual, family, and community levels [1,7]. In this study, we focused on the individual level of social capital, which inheres in the density or closure of community social networks. This level of social capital is recognized as a key predictor of older populations’ health [1,2,3,4,5], which can be used by older adults to preserve social supportive resources and pursue their individual or collective interests [12,13,14].

Community social capital is a multifaceted concept that can be divided into two categories, namely, cognitive and structural social capital [1]. Cognitive social capital is relatively subjective, and consists of the norms, values, and beliefs that influence people’s participation in society [7]. It is often measured by trust and reciprocity among neighbors [1]. Structural social capital is relatively objective, and reflects social interactions among people, which are often organized by formal organizations in the community. Structural social capital is often measured through organization memberships and social participation [7]. Family social capital is also considered as a multidimensional concept, which can be measured by both structural components and social support, interaction, and relationship in the family system [15]. Compared with the structural components of family social capital (e.g., the number of children), family network and support components (e.g., quality of family relationship and family support) had stronger impacts on the health outcomes among older adults [10,11,15]. Therefore, we examined family social capital from a family network support perspective in the present study. 

### 1.2. Determinants of Community Social Capital 

Community social capital is influenced by both microlevel determinants (e.g., income, education and health) and macrolevel determinants (e.g., income inequality and national cohesion). Microlevel determinants seem to be more influential for all of the dimensions of community social capital. Specifically, individuals’ socioeconomic status (i.e., income and education) is the most important determinant of community social capital [16]. 

As discussed previously, few studies have examined the multidimensional construct of social capital comprehensively. Instead, a range of indicators have been adopted to represent the levels of community social capital. For example, interpersonal trust, general trust, institutional trust, reciprocity among neighbors, and the sense of belonging are used to assess cognitive social capital [16,17,18,19,20]. Specifically, individuals with higher incomes and more education have higher levels of interpersonal trust [17,18,19]. Age, marital status, and religiosity are positively associated with both general and institutional trust [18,21]. Education tends to increase the likelihood of reciprocity, and age and general health were found to be positively associated with trust and the sense of belonging to local communities [20]. However, findings on the relationship between education, income, gender, and institutional trust are inconsistent [16,18]. The inconsistent findings might have to do with the lack of consensus on the operationalization of social capital, as well as the cultural and historical contexts from which samples have been drawn.

With regard to structural social capital, the number of organization memberships, social participation, volunteering, and civic participation are common adopted indicators [16,21,22,23]. Specifically, individuals who are older, male, and employed are likely to have more organization memberships, as are those who have higher income and education [16]. Ethnicity, marital status, and health are the most important determinants of social participation [22,24]. Furthermore, education, income, and health are the most important determinants of volunteering in later life [23,25]. Finally, higher income and education foster higher levels of civic participation [16]. In addition, men tend to have significantly higher levels of civic participation than women [21]. 

The majority of relevant studies have been conducted in Western contexts. Variations in levels of social capital by sociodemographic characteristics, socioeconomic status, and health status may be partly explained by variations in individuals’ health and financial resources, social status, time, and skills, which can further affect social involvement in the local community and society as a whole. These indicators tended to influence some components of social capital. However, few indicators were found to affect all of the components of social capital [18]. We argue that social capital cannot be observed directly. This concept should be treated as a latent construct, which are manifested by a range of observed variables [26]. Furthermore, microlevel determinants might have different impacts on different dimensions of social capital (i.e., cognitive and structural dimensions). Therefore, they should be studied separately while considering their specific cultural and historical contexts. In particular, most studies tend to depend on a single health indicator to assess the effect of health on social capital. Different components of health (e.g., activities of daily living (ADLs) and instrumental activities of daily living (IADLs)) might play different roles in influencing cognitive social capital and structural social capital. 

### 1.3. Population Aging and Family Social Capital in China

China is one of the most rapidly aging countries in the world. The number of adults 65 or older in China increased from 118.9 million in 2010 to 223.0 million in 2015 [27]. The proportion of older adults in the total population was 8.92% in 2010 and 10.47% in 2015. Given the rapid urbanization taking place in China, more and more older Chinese adults will be living in urban areas. 

How can China meet the long-term care needs of its rapidly aging population? This will be a great challenge for both Chinese policy makers and professionals in the next few decades. Family is the most important source of old-age care in China. Unlike the individualist West, China has a unique culture of Confucianism. Filial piety, a key concept of Confucianism, refers to older adults being well-respected and cared for by families and children. This is the foundation of cultural values as well as the affective bond in Chinese families [28]. However, traditional living arrangements for older adults have undergone a great transition. The majority of older Chinese lived with their adult children in the 1980s. However, the proportion of older adults who lived alone or with their spouse reached 22.84% in 2000 and 31.76% in 2010 [29]. Modernization and rural-to-urban migration have also weakened family support resources for older adults, especially in terms of instrumental support.

In a word, the traditional family-based old-age care system has undergone great challenges. As the demand for long-term care will increase dramatically in the next half century, experts should consider how to build more sustainable support systems for older populations in China. Community social capital not only helps older adults achieve aging in place, but can also be considered a preventive strategy for sustaining the welfare of older adults [1,2,7]. Fostering community social capital is also consistent with national policy development in China. In the recent seventh session of 13th National People’s Congress Standing Committee (December 2018), the Chinese government decided to extend the term of office of neighborhood committees from three years to five years. In doing so, neighborhood committees would have more stable teams to strengthen the functions of community and provide better quality of service for older residents in the communities. Under such circumstances, community social capital would play an important role in the future development of national long-term care systems in China. 

In this study, family social capital is considered a key determinant of community social capital in Chinese contexts. However, the interplay between family and community social capital has been largely unstudied among older populations in China. On the one hand, family is not only the preferred source or support for older adults, but also plays an important role in helping elders find meaning in life and enhancing their self-esteem [30]. Obedience and respect from offspring and family harmony are important indicators of success of life in older age [28]. Therefore, we consider family the strong tie for older adults, and community the weak tie. Older adults with good-quality family social capital might be more confident and willing to participate in informal exchanges among neighbors and friends in the communities. Poor family social capital, in contrast, might make older adults feel as though they are “losing face” and decrease the likelihood of trust, reciprocity among neighbors, and the sense of belonging to local communities. On the other hand, compared to informal exchanges among neighbors, older adults are more likely to have wider social connections in social involvements that are organized by formal organizations, such as clubs, volunteering, and civic participation. Therefore, family social capital might not affect older adults’ structural social capital levels. 

Therefore, based on social capital theory and the literature reviewed here, we investigated individual-level social determinants of cognitive and structural social capital among older adults in urban China, with a particular emphasis on health and family social capital. 

## 2. Materials and Methods 

### 2.1. Sampling

The Department of Social Work at Renmin University of China conducted a cross-sectional community survey in the city of Suzhou in the winter of 2015. Suzhou is a city in Jiangsu province, China. The sample was derived from 16 streets of Gusu District, which is the central urban area of Suzhou. This district shares many common features of naturally occurring retirement communities (NORC). There were approximately 949,600 people living in Gusu District in 2015 (Gusu District Government, 2016). The percentage of the residents 60 or older was approximately 25%, and the proportions in the 16 streets were similar. This figure is around 1.6 times higher than the national average in 2015 (16%; National Bureau of Statistics of China, 2016). Gender, marital status, education level, and income were all similar among all 16 streets. Old and small buildings are the main building styles in these communities. The majority of local residents owned their real estate properties and have lived in the communities for more than 10 years. As one of the most economically developed areas in China, Suzhou governments provided a range of community-based services for the local older residents, including but not limited to food delivery, home cleaning, health education, medical care, clubs, and volunteering. The levels of social capital (e.g., social trust) tend to be higher among older residents living in NORC than those living in communities with relatively higher proportions of migrant populations. Therefore, the above characteristics of Gusu District make it suitable for examining the determinants of cognitive and structural social capital.

A quota sampling technique was used to recruit adults 60 or older living in the 16 streets. Quota sampling is recognized as a non-probability sampling method [31]. Quota sampling accounts for population proportions (e.g., age and gender proportions), and can be used when it is not possible to implement probability sampling in the field. Given that the community is the smallest political division in China, one or two communities were selected from each of the 16 streets according to referrals from the local committee on aging and the community center. At the next steps, the researchers recruited respondents through the local committee on aging and community center referrals. Respondents who met the following conditions were included: (a) the respondent had local household registration status in Suzhou, (b) the respondent’s age was 60 or older, (c) the respondent had lived in the local community of one of the 16 streets for at least half a year in the past 12 months, and (d) the respondent had adequate cognitive capacity to complete the survey [32]. In order to enhance the representativeness of the sample, age and gender ratios among the selected respondents were similar, with those of the local representative sample from the most recent sixth national census [31]. 

Trained interviewers conducted via face-to-face interviews at respondents’ homes and the offices of the local community centers. The survey asked about respondents’ demographic characteristics, socioeconomic status, physical health, mental health, and social capital. A total of 456 respondents completed the survey successfully. The response rates were greater than 90% in all the communities.

### 2.2. Measurement

#### 2.2.1. Latent Variables

Based on the theory of social capital and the literature, we constructed two latent variables for community social capital that were not directly observed, but could be reflected and examined by a set of observed variables [26]. One measured cognitive social capital, and the other measured structural social capital. All of the quantitative measures of social capital were selected from the World Bank’s social capital questionnaire and the Short Social Capital Assessment tool [33,34]. While the former questionnaire has been frequently adopted in social capital studies conducted in Chinese communities, the latter assessment tool is designed to assess the cognitive and structural components of social capital in low and middle-income countries [7,33,34].

Cognitive social capital was measured with four items that represented the four aspects: trust in the local community, willingness to cooperate with others, feelings of belongingness, and perceptions of others’ helpfulness [33,34]. Participants responded to four statements: (a) “The majority of local residents living in this community can be trusted” (trust in the local community), (b) “Local residents care about both their benefits and others’ interests” (willingness to cooperate with others), (c) “Local community is a big family and consider yourself as a member of the big family” (feelings of belongingness), and (d) “Local residents help one another out” (perceptions of others’ helpfulness). Responses used a five-point Likert-type scale. For items (a), (b), and (c), one = strongly disagree, three = neutral, and five = strongly agree; for item (d), one = never helps, three = helps sometimes, and five = always helpful. The higher the score, the higher the cognitive social capital [33].

Structural social capital was measured with four items that represented the four aspects of organization memberships, social participation, volunteering, and citizenship activities [33,34]. Participants responded to four statements: (a) whether they were a member of the following organizations: political parties, women’s groups, neighborhood committees, labor unions, sports clubs, community associations, charitable organizations, credit groups, or religious groups (organization memberships), (b) “In the past 12 months, how frequently have you participated in activities organized by the above formal organizations on average?” (social participation), (c) “In the past month, how many hours per week have you spent on volunteering activities organized by the above formal organizations on average?” (volunteering), and (d) “Did you collaborate with other local residents to cope with a common issue or problem in the past year?” (citizenship activities). We recoded responses to item (a) as a binary variable (zero = no, one = yes), and summed scores represented participants’ number of organization memberships. Item (b) used a six-point scale (one = never participate, four = one to three times per month, six = more than twice per week). Responses to item (c) were further recoded as a dichotomous variable (zero = did not volunteer in the past month (i.e., zero volunteering hours), one = volunteered in the past month (i.e., more than 0 h)). Item (d) was also a dichotomous variable (zero = no, one = yes). The higher the score, the higher the structural social capital. 

#### 2.2.2. Determinants

The variables included sociodemographic variables such as age, gender, marital status, education level, and income; indicators of living status, such as how many children the respondents had and whether they lived alone; and indicators of health, such as ADLs, IADLs, SRH, and physical health. Finally, we added family social capital in the final model. 

Age was measured in years. Gender, marital status, living alone, and education level were binomial variables (one = female; one = married; one = yes; one = primary school level or higher). Respondents were asked about their monthly household income. The respondents were asked to report the number of their alive sons and daughters.

ADLs measured older adults’ dependence when walking, eating foods, dressing, washing their face, brushing their teeth, bathing, going up and down stairs, getting out of a chair and bed, going to the toilet, and controlling the bladder and bowels [35]. We summed the scores for the 10 items to represent ADLs (range = 0–100). The lower the score, the greater the dependence in ADLs. Regarding IADLs, the respondents were asked whether they had difficulty performing the following tasks: meal preparation, doing housework, managing finances, managing medications, using telephone, shopping, and using public transportation [36]. Responses were recoded on a three-point Likert scale (zero = great difficulty; one = some difficulties, assistance is needed; two = no difficulty). Summed scores were used to represent the levels of IADLs, with a range from zero to 14. The lower the score, the greater the dependence on IADLs. To examine respondents’ SRH, we asked a single question: “What do you think about your health status?” We recoded SRH as a binary variable (zero = fair/poor/very poor, one = excellent/good). Physical health was measured with the six most common chronic diseases among older adults in China. Respondents answered yes or no for each disease. We recoded the responses as binary variables (zero = no, one = yes), and used the summed scores to represent respondents’ physical health.

Finally, we used the four-item Multidimensional Scale of Perceived Social Support [37] to measure family social capital (Cronbach’s α = 0.917). The respondents were asked whether they agreed with the following four statements: (1) My family members are willing to help me when necessary; (2) I can receive emotional support from my family members; (3) I can discuss important issues with my family members; (4) My family members are willing to help me in decision making regarding important issues. Respondents answered on a five-point scale (zero = very disagree, two = fair, four = very agree). The scores were summed up to represent the quality of family social capital. The higher the score, the better the quality of family social capital. 

### 2.3. Data Analysis

We first conducted a descriptive analysis to calculate individual characteristics and other variables. Multiple indicators and multiple courses (MIMIC) from the perspective of structural equation modeling (SEM) was conducted to examine the social determinants of cognitive social capital and structural social capital. This approach has a few methodological merits. First and foremost, SEM allows the researchers to specify latent variables of social capital with different regression coefficient estimates of relationships between latent variables and the corresponding factor indicators (i.e., observed variables) [38]. Furthermore, SEM enables the researchers to not only account for measurement errors in the model, but also use a number of fit indexes to evaluate model fit [26]. 

The two-step analysis procedure is as follows. First, we used confirmatory factor analysis to construct two latent variables: cognitive and structural social capital. Factor loadings represented the relationships between the factor indicators and latent variables. Second, MIMIC was conducted to test the heterogeneity of the effects of the covariates on the constructs of cognitive and structural social capital. We used the fit indices below to test the model fit: the chi-square test statistic, root mean square error of approximation (RMSEA), comparative fit index (CFI), Tucker–Lewis index (TLI), and weighted root mean square residual (WRMR) [26]. Mplus 7.0 (Muthén & Muthén, Los Angeles, CA, USA) was used for statistical analyses. Since volunteering and citizenship activity (two-factor indicators of structural social capital) are binary variables, we used diagonally weighted least squares (WLSMV) as the estimator [39]. This estimator is designed for conducting SEM with ordinal and categorical variables. Finally, the missingness for all of the variables was below 5%. We used listwise deletion to handle missing data; the final sample size was 421.

## 3. Results

### 3.1. Descriptive Statistics

Table 1 summarizes the descriptive statistics for the data. Men made up 45.2% of the sample, and women made up 54.8%. The average age of the participants was 70.1 [standard deviation (SD) = 7.4]. A total of 75% of the respondents were married. Of the respondents, 34.9% had a primary school education or less, and 67.4% had a secondary school education or higher. The monthly income of 31.6% of respondents was less than 3000 RMB (434 USD). The majority of respondents (80.0%) were not religious. In addition, 82.7% of the respondents lived with others. Almost half of the respondents (51.1%) rated their health as good or very good. The mean scores of ADLs and IADLs were 98.99 (SD = 4.63; range = 0–100) and 13.65 (SD = 4.63; range = 0–14), respectively. In other words, 90.6% of the respondents had no limitations in ADLs, and 87.9% had no difficulty in performing IADL tasks. Respondents’ average number of children was 1.89 (1.07), and their mean number of diseases was 1.21 (SD = 1.03). Over average, 82.5% of the respondents reported a good quality of family social capital. 

### 3.2. Measurement Model of Cognitive and Structural Social Capital

First, we tested the measurement model. There were two latent constructs for social capital (cognitive and structural social capital), and four indicators for each construct. The estimates of fit indices indicated that the model adequately fit the data (χ^2^ (19) = 28.100, *p* = 0.0815; RMSEA = 0.032, CFI = 0.985, TLI = 0.978, and WRMR = 0.618). Standardized factor loadings ranged from 0.437 to 0.776 for cognitive social capital and from 0.606 to 0.731 for structural social capital. 

### 3.3. Results of MIMIC

Second, we used a MIMIC model to examine the effects of various determinants on cognitive and structural social capital. We added sociodemographic characteristics, health status, financial status, living arrangement, and family social capital as covariates. The model test statistics were as follows: χ^2^ (91) = 90.985, *p* = 0.4807. The nonsignificant chi-square estimate indicated a covariance matrix that was consistent with the covariance matrix of the sample [26]. The model fit indices indicated that the model adequately fit the data (RMSEA = 0.000, CFI = 1.000, TLI = 1.000, WRMR = 0.656). Family social capital, IADLs, and income were influential determinants of cognitive social capital: family social capital, β (SD) = 0.141 (0.025), *p* < 0.001; IADLs, β (SD) = 0.039 (0.015), *p* < 0.01; income, β (SD) = 0.024 (0.009), *p* < 0.01. Moreover, older women had more cognitive social capital than their male counterparts: β (SD) = 0.096 (0.038), *p* < 0.05. All of the other determinants were not significant predictors of cognitive social capital, which all had values of *p* > 0.05.

**Figure 1:**
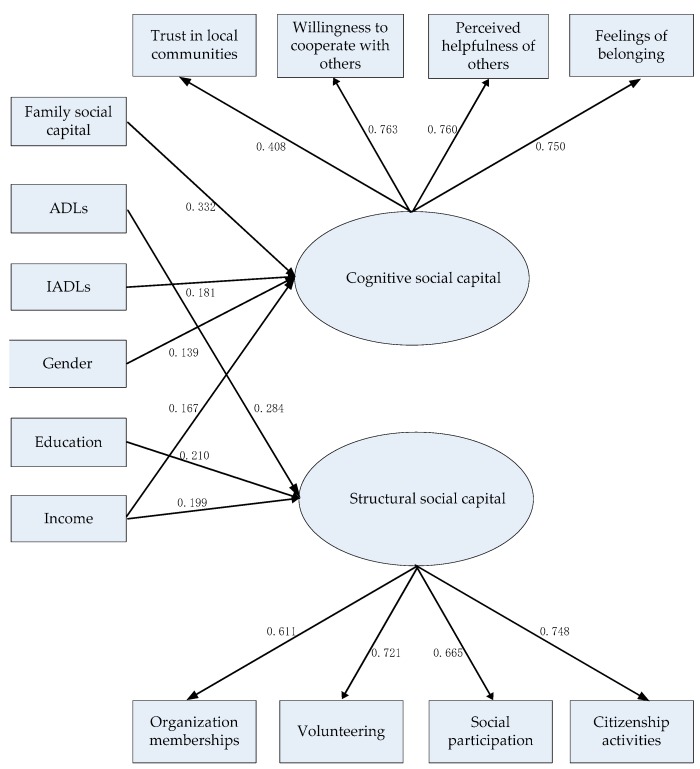
Final model of the determinants of cognitive and structural social capital. Standardized coefficients are reported. All of the estimates were statistically significant at the 0.05 level (two-tailed). Nonsignificant associations are not presented for the sake of simplicity. ADLs = activities of daily living. IADLs = instrumental activities of daily living.

ADLs, education, and income were significant determinants of structural social capital: ADLs, β (SD) = 0.050 (0.018), *p* < 0.01; education, β (SD) = 0.476 (0.155), *p* < 0.01; income, β (SD) = 0.077 (0.026), *p* < 0.01. The older the respondents, the less structural social capital they had: β (SD) = −0.017 (0.009), *p* < 0.01 (see Figure 1). All of the other determinants, including family social capital and IADLs, were not significant predictors of structural social capital, which all had values of *p* > 0.05.

## 4. Discussion

The relationship between socioeconomic status and social capital varies across different cultures and countries. Empirical evidence on the determinants of social capital in the Eastern Asian contexts is limited. The present study is one of the first attempts to use family social capital and health indicators to explain variations in the latent constructs of cognitive and structural social capital among older populations in an urban Chinese context. The findings not only provide new evidence to social capital theory from a Chinese perspective, but also have important implications for future policy and intervention development around healthy aging. 

Our findings confirm that eight factor indicators measure two latent variables. These two latent constructs—cognitive and structural social capital—are consistent with the social capital framework. Older adults with more cognitive social capital tend to report more social trust and reciprocity in local communities. In contrast, older adults with more structural social capital have more organization memberships, more frequent social participation, and are involved in more volunteering and citizenship activities. These two latent constructs provide more accurate and comprehensive tools for assessing social capital that could be used in future social work interventions to promote social capital in Chinese contexts. 

Consistent with previous studies, income was an influential determinant of both cognitive and structural social capital [17]. Individuals with higher incomes might be more optimistic than their counterparts, which leads to higher levels of trust, reciprocity, and group memberships. Poverty, in contrast, tends to foster distrust of others and society as a whole. It could also decrease one’s likelihood of being a member of an organization. Furthermore, the findings implied that education was associated with structural social capital only. The consequences of education include social status and adherence to social norms. Education systems provide people with additional access to social connections and foster social values of collaboration [17,40]. Education systems also help people be open-minded and develop knowledge and skills for basic social interactions [18]. These factors increase individuals’ participation in community activities. However, the finding showed that levels of cognitive social capital do not vary across older adults with different educational backgrounds. We conducted a sensitivity analysis by using different cutoff points of education, and generated similar results. The study of Halman and Luijkx [18] also found that education was not associated with interpersonal trust. The nonsignificant findings might partially be because less than 10% of the respondents had completed a college education. Future studies with larger national representative samples are needed to further examine the effect of education on cognitive social capital, and whether such association vary by cohorts. Furthermore, older women and men, and those with different economic status and educational background, tend to participate in different types of social and civic activities [16,21]. It is recommended that future studies examine the above issue and further test the social determinants of social capital through consideration of the contexts of different community organizations. 

Furthermore, our findings supported the social selection model [8]. Consistent with previous studies, health was found to be significantly associated with social capital [20,22,24]. Our findings add new evidence that ADLs are more important for structural social capital, whereas IADLs are more important for cognitive social capital. This might be because ADL limitations could decrease the likelihood of participating in social activities organized by formal organizations. On the other hand, capacities of performing IADL tasks help older adults not only live independently in the community, they also foster informal reciprocity among neighbors and a sense of belonging to local communities. We regressed structural social capital on IADLs, and the results showed that the above association was statistically significant. However, the association became nonsignificant in the final model, after controlling for other determinants. This means that the above association can be explained by other determinants such as ADLs and socioeconomic status. We conducted similar analysis to examine the association between ADLs and cognitive social capital, and reached similar conclusions. Future longitudinal studies are needed to examine the interplay between health trajectories and social capital trajectories. Such findings can provide more in-depth understandings of how health and social capital interact with each other over the life course. 

Family social capital was the most influential determinant of cognitive social capital, even after we controlled for sociodemographic characteristics, socioeconomic status, and health variables. The findings indicate that family social capital plays a crucial role in older adults’ social involvements in local communities. Good-quality family social capital fulfills cultural expectations and enhances self-esteem, which (a) makes adults more likely to trust other residents in the local community and become involved in reciprocal exchanges with neighbors, and (b) fosters a sense of belonging in local communities. However, the correlation analysis showed that the association between family social capital and structural social capital was only marginally significant at the 0.1 level. This association became statistically nonsignificant when we controlled for other variables. This means that the relationship between family social capital and structural social capital can be explained by other third variables (i.e., age, socioeconomic status, and ADLs). Future studies are needed to examine the effects of family social capital on social participation in different social organizations. It is also recommended that future studies test regional disparities in terms of the social determinants of social capital in older age.

The present study has the following implications for policy and intervention. Organization membership and citizenship activity are Western-oriented concepts, which reflect individuals’ civic engagements in formal organizations to pursue individual and collective interests. While older Chinese adults are still involved in informal groups, the establishment of measurement models of social capital supports that formal organizations are becoming more important in urban Chinese communities. Older adults share knowledge, information, and social resources in both informal and formal organizations in Suzhou. Policy makers should put great emphasis on promoting the role of formal organizations in enhancing older residents’ independence, autonomy, meaning in life, and senses of attachment and belonging in their communities. Specifically, the latent constructs of cognitive social capital and structural social capital can be used in evaluation programs for community-building programs. Different types of social organizations should be established in the communities so that older adults have adequate access to actively participate in community activities. Neighborhood committees and community social workers could help older adults address their collective interests through promoting volunteering programs and citizenship activities. Social capital programs can be used to meet older adults’ personal and collective interests from a community perspective. To some extent, this is more cost-effective and efficient than programs that provide direct services to individuals. In other words, social capital would play an important role in developing prevention programs targeting the health among older populations, and reducing financial burden in the long-term-care systems in China. 

Additionally, older adults of low socioeconomic status and poor health conditions deserve attention from both policy makers and designers of interventions. While this specific population tends to report lower levels of social capital than their counterparts, they might also be the groups that benefit most from the promotion of social capital. In this case, their levels of social capital should be further evaluated in comprehensive needs assessments. Such information is valuable for both anti-poverty policies and long-term care policies. In particular, given the health benefits of volunteering and social participation in later life, interventions that aim to promote structural social capital should pay particular attention to social needs and potential barriers among less educated older adults. Motivational prompts and engagement in team building should be used to explain the health benefits of volunteering, and promote altruism and goal setting [41,42].

Moreover, given the different impacts of ADLs and IADLs on community social capital, it is recommended that community social capital can be promoted through rigorously improving the physical environment, facilitating information diffusion and enhancing community service utilization. Furthermore, the findings suggest that interventions that aim to promote family social capital might improve older adults’ levels of cognitive social capital (i.e., individuals’ subjective evaluations of trust and reciprocity in their local communities). However, such interventions might not have significant impacts on their levels of structural social capital (e.g., social participation and group membership). On the other hand, older adults with low levels of family social capital might also experience low levels of community social capital, which means that they could experience double jeopardy. Therefore, the interplay between family social capital and community social capital should not be ignored in future policy strategies and intervention designs. 

Despite these strengths, this study has a few limitations. First, the nature of the cross-sectional data did not allow us to examine the direction of causality between family social capital, health status, and community social capital. Based on social capital theory and the relevant literature, we discussed the theoretical rationale for the proposed model. Second, the present study focused on individual-level determinants of social capital in later life. Future studies need to examine macro-level determinants, such as the role of welfare systems, education systems, and labor markets. Third, the data were not randomly selected. As discussed previously, the majority of old urban communities are preserved in the Gusu district of Suzhou city, which leads to relatively high levels of community social capital through strong social trust, informal reciprocity, and the provision of aged care service. The findings of this study should be considered illustrative of older adults living in NORC in urban China. Finally, the latent constructs of social capital should be further tested in other community contexts (e.g., rural areas and small cities). For example, future social capital studies should be conducted in Chinese communities with higher proportions of migrant populations. Low levels of social trust and reciprocity might be identified in these communities. Findings based on NORC can be used as important references for community development programs in other urban Chinese communities.

## 5. Conclusions

This study establishes two latent constructs of cognitive social capital and structural social capital in urban Chinese contexts. Social capital can be developed through enhancing older adults’ independence, autonomy, reciprocity, and sense of belonging to local communities. Neighborhood committees and community social workers should enrich meanings in later life, and address local residents’ collective interests through a range of volunteering programs and citizenship activities. Physical environment, information diffusion, and community service utilization could also be vital for the development of community social capital. 

The findings suggest that latent constructs of community social capital can be used as a more comprehensive screening and evaluation tool in future social capital policy and intervention designs. Family social capital, health, and socioeconomic status are important indicators to screen populations who are at risk of low levels of community social capital. Older adults with low levels of family social capital and community social capital might experience double jeopardy. Furthermore, the social needs and potential barriers for less educated older adults deserve particular attention from designers of social interventions aiming to promote structural social capital in later life. Promoting the quality of family social capital also helps older adults enhance their trust and reciprocity with neighbors and foster their sense of belonging in local communities. Motivational prompts and engagement in team building should be used to encourage older adults to be involved in volunteering and other social and collective activities. 

## Figures and Tables

**Table 1:** Characteristics of the sample (*N* = 456).

Characteristic	*n* (%)	M (SD)
Age		70.1 (7.4)
Gender		
Male	206 (45.2)	
Female	250 (54.8)	
Marital status		
Married	342 (75.0)	
Other	114 (25.0)	
Education		
Illiterate	70 (15.4)	
Primary school or higher	377 (82.7)	
Income		
RMB 3000 or less	143 (31.6)	
RMB 3001–5000	100 (21.9)	
RMB 5001 or more	209 (45.9)	
Living alone		
Yes	79 (17.3)	
No	377 (82.7)	
Self-rated health		
Good/very good	233 (51.1)	
Very poor/poor/fair	222 (48.7)	
Number of children		1.89 (1.07)

Notes: 100 RMB = 14.48 USD; *N* = number; M = Mean; SD = standard deviation.
